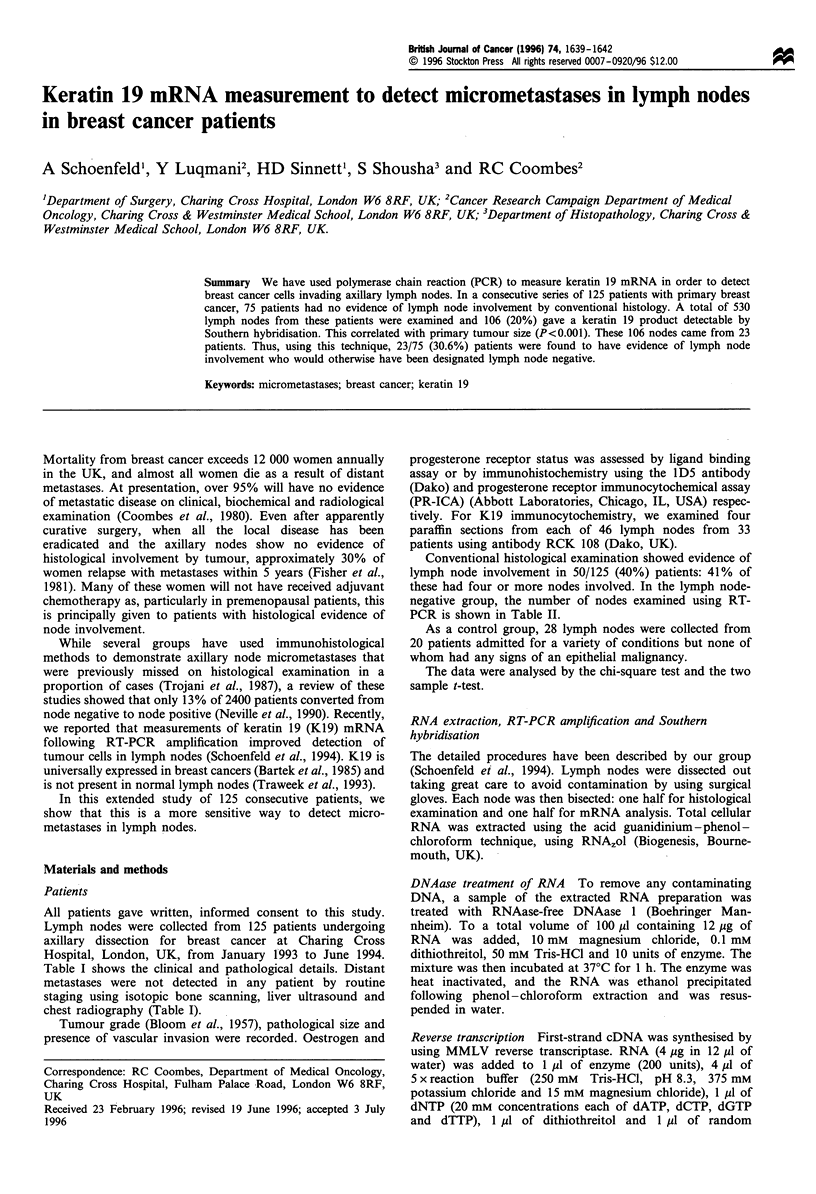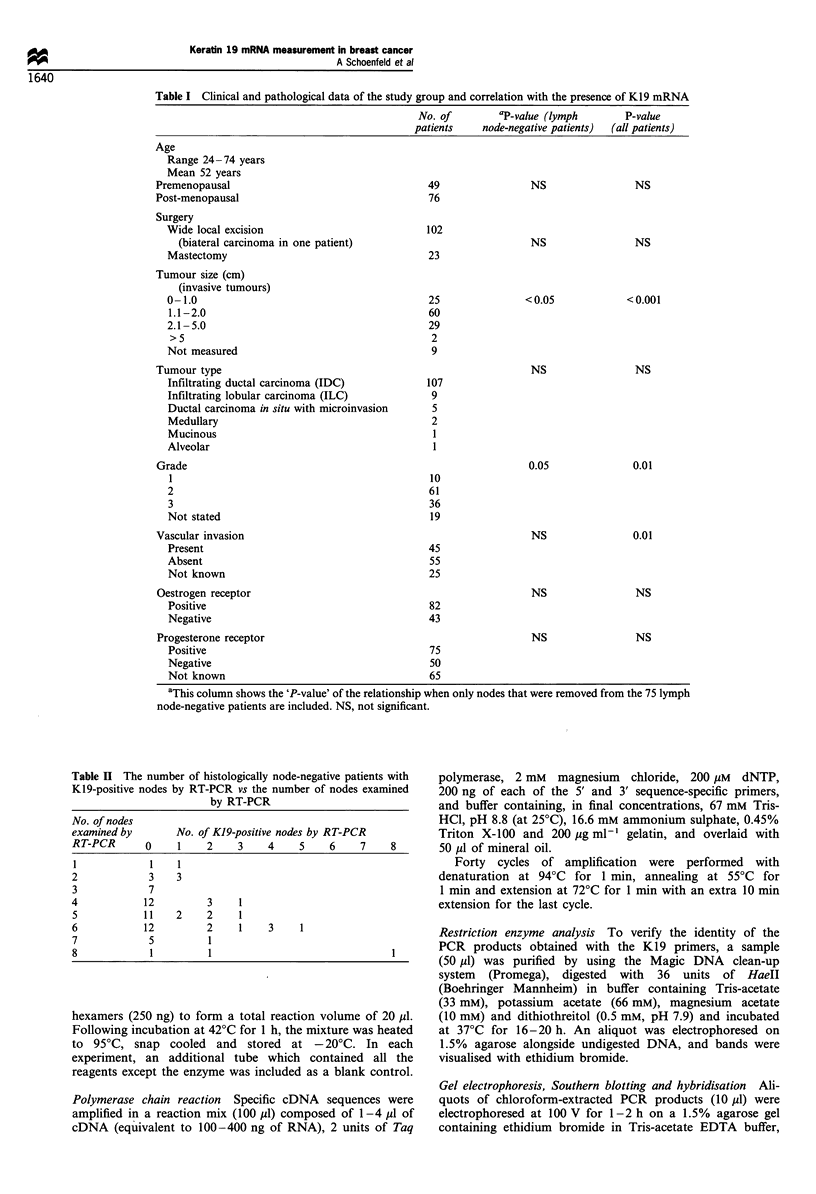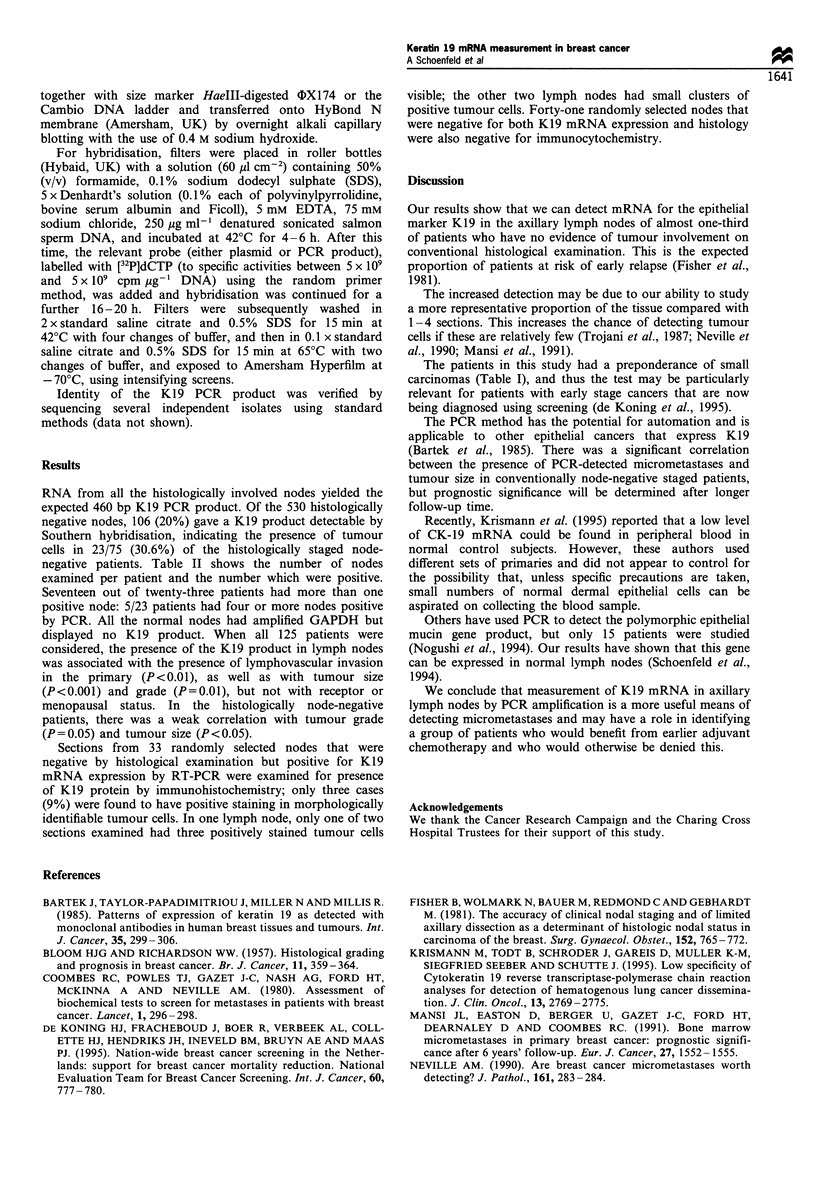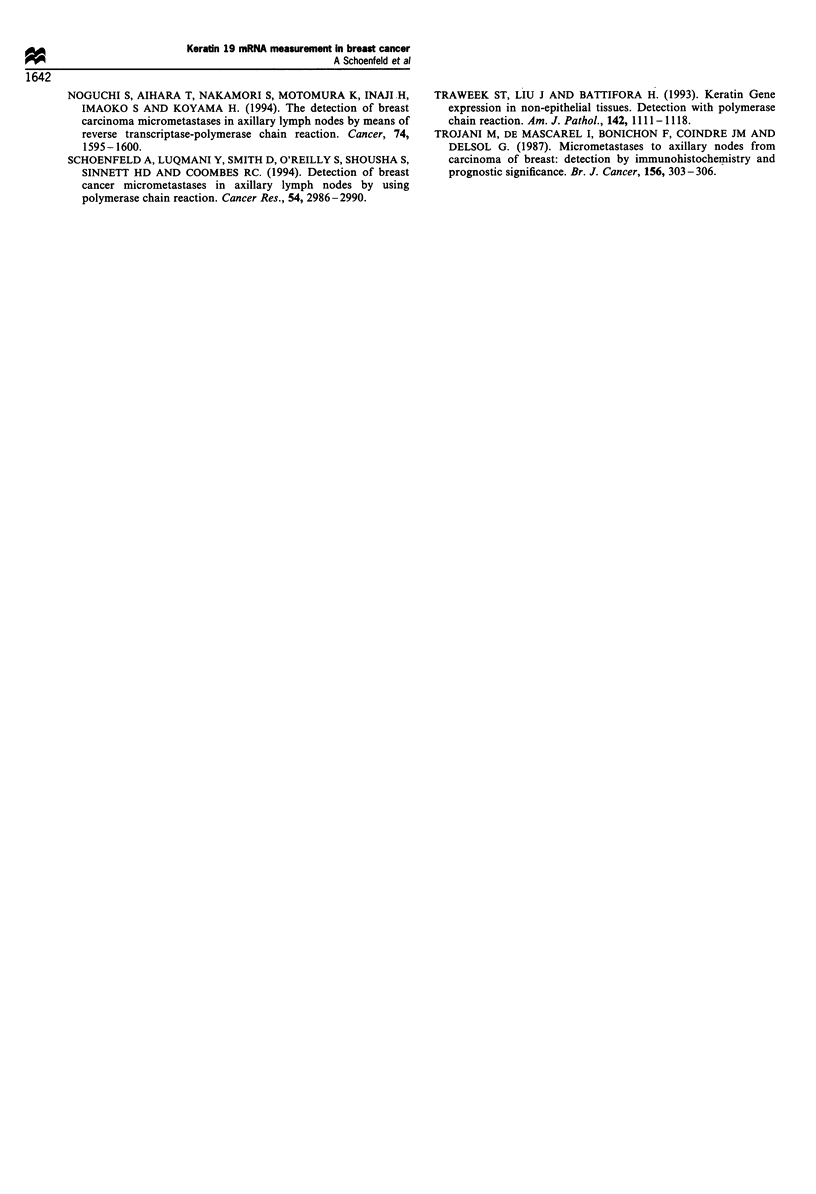# Keratin 19 mRNA measurement to detect micrometastases in lymph nodes in breast cancer patients.

**DOI:** 10.1038/bjc.1996.601

**Published:** 1996-11

**Authors:** A. Schoenfeld, Y. Luqmani, H. D. Sinnett, S. Shousha, R. C. Coombes

**Affiliations:** Department of Surgery, Charing Cross Hospital, London, UK.

## Abstract

We have used polymerase chain reaction (PCR) to measure keratin 19 mRNA in order to detect breast cancer cells invading axillary lymph nodes. In a consecutive series of 125 patients with primary breast cancer, 75 patients had no evidence of lymph node involvement by conventional histology. A total of 530 lymph nodes from these patients were examined and 106 (20%) gave a keratin 19 product detectable by Southern hybridisation. This correlated with primary tumour size (P<0.001). These 106 nodes came from 23 patients. Thus, using this technique, 23/75 (30.6%) patients were found to have evidence of lymph node involvement who would otherwise have been designated lymph node negative.


British Journal of Cancer (1996) 74, 1639-1642
© 1996 Stockton Press All rights reserved 0007-0920/96 $12.00

### Keratin 19 mRNA measurement to detect micrometastases in lymph nodes in breast cancer patients

A Schoenfeld[1], Y Luqmani[2], HD Sinnett[1], S Shousha[3] and RC Coombes[2]

[1]*Department of Surgery, Charing Cross Hospital, London W6 8RF, UK;* [2]*Cancer Research Campaign Department of Medical Oncology, Charing Cross & Westminster Medical School, London W6 8RF, UK;* [3]*Department of Histopathology, Charing Cross & Westminster Medical School, London W6 8RF, UK.*

**Summary** We have used polymerase chain reaction (PCR) to measure keratin 19 mRNA in order to detect breast cancer cells invading axillary lymph nodes. In a consecutive series of 125 patients with primary breast cancer, 75 patients had no evidence of lymph node involvement by conventional histology. A total of 530 lymph nodes from these patients were examined and 106 (20%) gave a keratin 19 product detectable by Southern hybridisation. This correlated with primary tumour size ($P<0.001$). These 106 nodes came from 23 patients. Thus, using this technique, 23/75 (30.6%) patients were found to have evidence of lymph node involvement who would otherwise have been designated lymph node negative.

**Keywords:** micrometastases; breast cancer; keratin 19

Mortality from breast cancer exceeds 12 000 women annually in the UK, and almost all women die as a result of distant metastases. At presentation, over 95% will have no evidence of metastatic disease on clinical, biochemical and radiological examination (Coombes *et al.*, 1980). Even after apparently curative surgery, when all the local disease has been eradicated and the axillary nodes show no evidence of histological involvement by tumour, approximately 30% of women relapse with metastases within 5 years (Fisher *et al.*, 1981). Many of these women will not have received adjuvant chemotherapy as, particularly in premenopausal patients, this is principally given to patients with histological evidence of node involvement.

While several groups have used immunohistological methods to demonstrate axillary node micrometastases that were previously missed on histological examination in a proportion of cases (Trojani *et al.*, 1987), a review of these studies showed that only 13% of 2400 patients converted from node negative to node positive (Neville *et al.*, 1990). Recently, we reported that measurements of keratin 19 (K19) mRNA following RT-PCR amplification improved detection of tumour cells in lymph nodes (Schoenfeld *et al.*, 1994). K19 is universally expressed in breast cancers (Bartek *et al.*, 1985) and is not present in normal lymph nodes (Traweek *et al.*, 1993).

In this extended study of 125 consecutive patients, we show that this is a more sensitive way to detect micrometastases in lymph nodes.

### Materials and methods

### *Patients*

All patients gave written, informed consent to this study. Lymph nodes were collected from 125 patients undergoing axillary dissection for breast cancer at Charing Cross Hospital, London, UK, from January 1993 to June 1994. Table I shows the clinical and pathological details. Distant metastases were not detected in any patient by routine staging using isotopic bone scanning, liver ultrasound and chest radiography (Table I).

Tumour grade (Bloom *et al.*, 1957), pathological size and presence of vascular invasion were recorded. Oestrogen and progesterone receptor status was assessed by ligand binding assay or by immunohistochemistry using the 1D5 antibody (Dako) and progesterone receptor immunocytochemical assay (PR-ICA) (Abbott Laboratories, Chicago, IL, USA) respectively. For K19 immunocytochemistry, we examined four paraffin sections from each of 46 lymph nodes from 33 patients using antibody RCK 108 (Dako, UK).

Conventional histological examination showed evidence of lymph node involvement in 50/125 (40%) patients: 41% of these had four or more nodes involved. In the lymph node-negative group, the number of nodes examined using RT-PCR is shown in Table II.

As a control group, 28 lymph nodes were collected from 20 patients admitted for a variety of conditions but none of whom had any signs of an epithelial malignancy.

The data were analysed by the chi-square test and the two sample *t*-test.

### *RNA extraction, RT-PCR amplification and Southern hybridisation*

The detailed procedures have been described by our group (Schoenfeld *et al.*, 1994). Lymph nodes were dissected out taking great care to avoid contamination by using surgical gloves. Each node was then bisected: one half for histological examination and one half for mRNA analysis. Total cellular RNA was extracted using the acid guanidinium-phenol-chloroform technique, using $RNA_zol$ (Biogenesis, Bournemouth, UK).

*DNAase treatment of RNA* To remove any contaminating DNA, a sample of the extracted RNA preparation was treated with RNAase-free DNAase 1 (Boehringer Mannheim). To a total volume of 100 $\mu$l containing 12 $\mu$g of RNA was added, 10 mM magnesium chloride, 0.1 mM dithiothreitol, 50 mM Tris-HCl and 10 units of enzyme. The mixture was then incubated at 37°C for 1 h. The enzyme was heat inactivated, and the RNA was ethanol precipitated following phenol-chloroform extraction and was resuspended in water.

*Reverse transcription* First-strand cDNA was synthesised by using MMLV reverse transcriptase. RNA (4 $\mu$g in 12 $\mu$l of water) was added to 1 $\mu$l of enzyme (200 units), 4 $\mu$l of 5 x reaction buffer (250 mM Tris-HCl, pH 8.3, 375 mM potassium chloride and 15 mM magnesium chloride), 1 $\mu$l of dNTP (20 mM concentrations each of dATP, dCTP, dGTP and dTTP), 1 $\mu$l of dithiothreitol and 1 $\mu$l of random

Correspondence: RC Coombes, Department of Medical Oncology, Charing Cross Hospital, Fulham Palace Road, London W6 8RF, UK
Received 23 February 1996; revised 19 June 1996; accepted 3 July 1996

Table I Clinical and pathological data of the study group and correlation with the presence of K19 mRNA

| | *No. of patients* | [a]*P-value (lymph node-negative patients)* | *P-value (all patients)* |
|---|---|---|---|
| Age | | | |
| Range 24–74 years | | | |
| Mean 52 years | | | |
| Premenopausal | 49 | NS | NS |
| Post-menopausal | 76 | | |
| Surgery | | | |
| Wide local excision | 102 | | |
| (biateral carcinoma in one patient) | | NS | NS |
| Mastectomy | 23 | | |
| Tumour size (cm) | | | |
| (invasive tumours) | | | |
| 0–1.0 | 25 | <0.05 | <0.001 |
| 1.1–2.0 | 60 | | |
| 2.1–5.0 | 29 | | |
| >5 | 2 | | |
| Not measured | 9 | | |
| Tumour type | | NS | NS |
| Infiltrating ductal carcinoma (IDC) | 107 | | |
| Infiltrating lobular carcinoma (ILC) | 9 | | |
| Ductal carcinoma *in situ* with microinvasion | 5 | | |
| Medullary | 2 | | |
| Mucinous | 1 | | |
| Alveolar | 1 | | |
| Grade | | 0.05 | 0.01 |
| 1 | 10 | | |
| 2 | 61 | | |
| 3 | 36 | | |
| Not stated | 19 | | |
| Vascular invasion | | NS | 0.01 |
| Present | 45 | | |
| Absent | 55 | | |
| Not known | 25 | | |
| Oestrogen receptor | | NS | NS |
| Positive | 82 | | |
| Negative | 43 | | |
| Progesterone receptor | | NS | NS |
| Positive | 75 | | |
| Negative | 50 | | |
| Not known | 65 | | |

[a]This column shows the '*P*-value' of the relationship when only nodes that were removed from the 75 lymph node-negative patients are included. NS, not significant.

Table II The number of histologically node-negative patients with K19-positive nodes by RT-PCR *vs* the number of nodes examined by RT-PCR

| *No. of nodes examined by RT-PCR* | *No. of K19-positive nodes by RT-PCR* 0 | 1 | 2 | 3 | 4 | 5 | 6 | 7 | 8 |
|---|---|---|---|---|---|---|---|---|---|
| 1 | 1 | 1 | | | | | | | |
| 2 | 3 | 3 | | | | | | | |
| 3 | 7 | | | | | | | | |
| 4 | 12 | | 3 | 1 | | | | | |
| 5 | 11 | 2 | 2 | 1 | | | | | |
| 6 | 12 | | 2 | 1 | 3 | 1 | | | |
| 7 | 5 | | 1 | | | | | | |
| 8 | 1 | | 1 | | | | | | 1 |

hexamers (250 ng) to form a total reaction volume of 20 $\mu$l. Following incubation at 42°C for 1 h, the mixture was heated to 95°C, snap cooled and stored at −20°C. In each experiment, an additional tube which contained all the reagents except the enzyme was included as a blank control.

*Polymerase chain reaction* Specific cDNA sequences were amplified in a reaction mix (100 $\mu$l) composed of 1–4 $\mu$l of cDNA (equivalent to 100–400 ng of RNA), 2 units of *Taq* polymerase, 2 mM magnesium chloride, 200 $\mu$M dNTP, 200 ng of each of the 5′ and 3′ sequence-specific primers, and buffer containing, in final concentrations, 67 mM Tris-HCl, pH 8.8 (at 25°C), 16.6 mM ammonium sulphate, 0.45% Triton X-100 and 200 $\mu$g ml$^{-1}$ gelatin, and overlaid with 50 $\mu$l of mineral oil.

Forty cycles of amplification were performed with denaturation at 94°C for 1 min, annealing at 55°C for 1 min and extension at 72°C for 1 min with an extra 10 min extension for the last cycle.

*Restriction enzyme analysis* To verify the identity of the PCR products obtained with the K19 primers, a sample (50 $\mu$l) was purified by using the Magic DNA clean-up system (Promega), digested with 36 units of *Hae*II (Boehringer Mannheim) in buffer containing Tris-acetate (33 mM), potassium acetate (66 mM), magnesium acetate (10 mM) and dithiothreitol (0.5 mM, pH 7.9) and incubated at 37°C for 16–20 h. An aliquot was electrophoresed on 1.5% agarose alongside undigested DNA, and bands were visualised with ethidium bromide.

*Gel electrophoresis, Southern blotting and hybridisation* Aliquots of chloroform-extracted PCR products (10 $\mu$l) were electrophoresed at 100 V for 1–2 h on a 1.5% agarose gel containing ethidium bromide in Tris-acetate EDTA buffer, together with size marker *Hae*III-digested ΦX174 or the Cambio DNA ladder and transferred onto HyBond N membrane (Amersham, UK) by overnight alkali capillary blotting with the use of 0.4 M sodium hydroxide.

For hybridisation, filters were placed in roller bottles (Hybaid, UK) with a solution (60 $\mu$l cm$^{-2}$) containing 50% (v/v) formamide, 0.1% sodium dodecyl sulphate (SDS), 5 × Denhardt's solution (0.1% each of polyvinylpyrrolidine, bovine serum albumin and Ficoll), 5 mM EDTA, 75 mM sodium chloride, 250 $\mu$g ml$^{-1}$ denatured sonicated salmon sperm DNA, and incubated at 42°C for 4–6 h. After this time, the relevant probe (either plasmid or PCR product), labelled with [$^{32}$P]dCTP (to specific activities between $5 \times 10^9$ and $5 \times 10^9$ cpm $\mu$g$^{-1}$ DNA) using the random primer method, was added and hybridisation was continued for a further 16–20 h. Filters were subsequently washed in 2 × standard saline citrate and 0.5% SDS for 15 min at 42°C with four changes of buffer, and then in 0.1 × standard saline citrate and 0.5% SDS for 15 min at 65°C with two changes of buffer, and exposed to Amersham Hyperfilm at −70°C, using intensifying screens.

Identity of the K19 PCR product was verified by sequencing several independent isolates using standard methods (data not shown).

### Results

RNA from all the histologically involved nodes yielded the expected 460 bp K19 PCR product. Of the 530 histologically negative nodes, 106 (20%) gave a K19 product detectable by Southern hybridisation, indicating the presence of tumour cells in 23/75 (30.6%) of the histologically staged node-negative patients. Table II shows the number of nodes examined per patient and the number which were positive. Seventeen out of twenty-three patients had more than one positive node: 5/23 patients had four or more nodes positive by PCR. All the normal nodes had amplified GAPDH but displayed no K19 product. When all 125 patients were considered, the presence of the K19 product in lymph nodes was associated with the presence of lymphovascular invasion in the primary ($P<0.01$), as well as with tumour size ($P<0.001$) and grade ($P=0.01$), but not with receptor or menopausal status. In the histologically node-negative patients, there was a weak correlation with tumour grade ($P=0.05$) and tumour size ($P<0.05$).

Sections from 33 randomly selected nodes that were negative by histological examination but positive for K19 mRNA expression by RT-PCR were examined for presence of K19 protein by immunohistochemistry; only three cases (9%) were found to have positive staining in morphologically identifiable tumour cells. In one lymph node, only one of two sections examined had three positively stained tumour cells visible; the other two lymph nodes had small clusters of positive tumour cells. Forty-one randomly selected nodes that were negative for both K19 mRNA expression and histology were also negative for immunocytochemistry.

### Discussion

Our results show that we can detect mRNA for the epithelial marker K19 in the axillary lymph nodes of almost one-third of patients who have no evidence of tumour involvement on conventional histological examination. This is the expected proportion of patients at risk of early relapse (Fisher *et al.*, 1981).

The increased detection may be due to our ability to study a more representative proportion of the tissue compared with 1–4 sections. This increases the chance of detecting tumour cells if these are relatively few (Trojani *et al.*, 1987; Neville *et al.*, 1990; Mansi *et al.*, 1991).

The patients in this study had a preponderance of small carcinomas (Table I), and thus the test may be particularly relevant for patients with early stage cancers that are now being diagnosed using screening (de Koning *et al.*, 1995).

The PCR method has the potential for automation and is applicable to other epithelial cancers that express K19 (Bartek *et al.*, 1985). There was a significant correlation between the presence of PCR-detected micrometastases and tumour size in conventionally node-negative staged patients, but prognostic significance will be determined after longer follow-up time.

Recently, Krismann *et al.* (1995) reported that a low level of CK-19 mRNA could be found in peripheral blood in normal control subjects. However, these authors used different sets of primaries and did not appear to control for the possibility that, unless specific precautions are taken, small numbers of normal dermal epithelial cells can be aspirated on collecting the blood sample.

Others have used PCR to detect the polymorphic epithelial mucin gene product, but only 15 patients were studied (Nogushi *et al.*, 1994). Our results have shown that this gene can be expressed in normal lymph nodes (Schoenfeld *et al.*, 1994).

We conclude that measurement of K19 mRNA in axillary lymph nodes by PCR amplification is a more useful means of detecting micrometastases and may have a role in identifying a group of patients who would benefit from earlier adjuvant chemotherapy and who would otherwise be denied this.

**Acknowledgements**
We thank the Cancer Research Campaign and the Charing Cross Hospital Trustees for their support of this study.

### References

BARTEK J, TAYLOR-PAPADIMITRIOU J, MILLER N AND MILLIS R. (1985). Patterns of expression of keratin 19 as detected with monoclonal antibodies in human breast tissues and tumours. *Int. J. Cancer*, **35**, 299–306.

BLOOM HJG AND RICHARDSON WW. (1957). Histological grading and prognosis in breast cancer. *Br. J. Cancer*, **11**, 359–364.

COOMBES RC, POWLES TJ, GAZET J-C, NASH AG, FORD HT, MCKINNA A AND NEVILLE AM. (1980). Assessment of biochemical tests to screen for metastases in patients with breast cancer. *Lancet*, **1**, 296–298.

DE KONING HJ, FRACHEBOUD J, BOER R, VERBEEK AL, COLLETTE HJ, HENDRIKS JH, INEVELD BM, BRUYN AE AND MAAS PJ. (1995). Nation-wide breast cancer screening in the Netherlands: support for breast cancer mortality reduction. National Evaluation Team for Breast Cancer Screening. *Int. J. Cancer*, **60**, 777–780.

FISHER B, WOLMARK N, BAUER M, REDMOND C AND GEBHARDT M. (1981). The accuracy of clinical nodal staging and of limited axillary dissection as a determinant of histologic nodal status in carcinoma of the breast. *Surg. Gynaecol. Obstet.*, **152**, 765–772.

KRISMANN M, TODT B, SCHRODER J, GAREIS D, MULLER K-M, SIEGFRIED SEEBER AND SCHUTTE J. (1995). Low specificity of Cytokeratin 19 reverse transcriptase-polymerase chain reaction analyses for detection of hematogenous lung cancer dissemination. *J. Clin. Oncol.*, **13**, 2769–2775.

MANSI JL, EASTON D, BERGER U, GAZET J-C, FORD HT, DEARNALEY D AND COOMBES RC. (1991). Bone marrow micrometastases in primary breast cancer: prognostic significance after 6 years' follow-up. *Eur. J. Cancer*, **27**, 1552–1555.

NEVILLE AM. (1990). Are breast cancer micrometastases worth detecting? *J. Pathol.*, **161**, 283–284.

NOGUCHI S, AIHARA T, NAKAMORI S, MOTOMURA K, INAJI H, IMAOKO S AND KOYAMA H. (1994). The detection of breast carcinoma micrometastases in axillary lymph nodes by means of reverse transcriptase-polymerase chain reaction. *Cancer*, **74**, 1595–1600.

SCHOENFELD A, LUQMANI Y, SMITH D, O'REILLY S, SHOUSHA S, SINNETT HD AND COOMBES RC. (1994). Detection of breast cancer micrometastases in axillary lymph nodes by using polymerase chain reaction. *Cancer Res.*, **54**, 2986–2990.

TRAWEEK ST, LIU J AND BATTIFORA H. (1993). Keratin Gene expression in non-epithelial tissues. Detection with polymerase chain reaction. *Am. J. Pathol.*, **142**, 1111–1118.

TROJANI M, DE MASCAREL I, BONICHON F, COINDRE JM AND DELSOL G. (1987). Micrometastases to axillary nodes from carcinoma of breast: detection by immunohistochemistry and prognostic significance. *Br. J. Cancer*, **156**, 303–306.